# Inflammatory Markers and Outcomes in Cardiovascular Disease

**DOI:** 10.1371/journal.pmed.1000147

**Published:** 2009-09-08

**Authors:** Leonard Kritharides

**Affiliations:** 1 Department of Cardiology Concord Repatriation General Hospital, Sydney South Western Area Health Service, University of Sydney, New South Wales, Australia; 2 Centre for Vascular Research University of New South Wales, Sydney, New South Wales, Australia

## Abstract

In a commentary on two new research studies in *PLoS Medicine*, Leonard Kritharides discusses the role of inflammatory markers in predicting cardiovascular outcomes and patients' responses to treatment.

**Linked Research Article**

This Perspective discusses the following new study published in *PLoS Medicine*:

Whiteley W, Jackson C, Lewis S, Lowe G, Rumley A, et al. (2009) Inflammatory Markers and Poor Outcome after Stroke: A Prospective Cohort Study and Systematic Review of Interleukin-6. PLoS Med 6(9): e1000145. doi:10.1371/journal.pmed.1000145

In a prospective cohort study of patient outcomes following stroke, William Whiteley and colleagues find that markers of inflammatory response are associated with poor outcomes. However, addition of these markers to existing prognostic models does not improve outcome prediction.

## Background and Research Questions

Inflammatory processes are clearly implicated in the aetiology of vascular disease and in its sequelae [1]. Atherosclerosis causes ischaemia and infarction by the chronic or acute occlusion of arteries, and inflammatory cells have been identified in atherosclerotic lesions. Systemic markers of inflammation such as interleukin-6 (IL-6), C-reactive protein (CRP), fibrinogen, and white cell count have previously been shown to be associated with increased risk of cardiovascular events in apparently healthy populations and patients with established vascular disease (e.g., [2],[3]), and in predicting the risk of, and outcome after, stroke (reviewed in [4]). Although inflammatory markers do improve prediction of cardiovascular events, their predictive value may be modest [5]. Others have argued that the use of receiver operating characteristic (ROC) curves and C-statistics to decide the value of biomarkers may underestimate their value in certain populations [6]. Recently, prediction of mortality was shown to be improved when combinations of biomarkers are used [7]. Because healthy populations with CRP over 2 mg/dl show reduced rates of fatal and nonfatal cardiovascular events in response to treatment with statin [8], it is clinically important to clarify the role of inflammatory markers in predicting outcomes and the response to treatment.

## Two Studies with Apparently Conflicting Messages

Two studies published in *PLoS Medicine*—one previously [9] and the other in this issue [10]—that investigate the predictive value of inflammatory markers in cardiovascular disease arrive at apparently conflicting conclusions. Sattar et al. investigated over 5,000 men and women who were aged 70–82 y and at high risk of suffering cardiovascular events as part of the PROSPER study [9]. In these individuals, who were randomised to placebo or treatment with pravastatin, IL-6, CRP, and fibrinogen all predicted cardiovascular death, and IL-6 in particular added significantly to conventional risk factors in predicting those suffering fatal myocardial infarction or fatal stroke. In contrast, inflammatory markers were weak predictors of nonfatal cardiovascular disease. These conclusions were unaffected by treatment with pravastatin. The implications are that the many studies pooling fatal and nonfatal cardiovascular outcomes may have inadvertently underestimated the true predictive power of inflammatory markers to predict fatal AU cardiovascular events.

Elevated inflammatory markers also predicted increased risk of noncardiovascular death in this population, suggesting these inflammatory markers are not necessarily atherosclerosis-specific. Unrecognised infection, chronic illness, and malignancy, all potentially predisposing to thrombosis, may play a role. The number of patients actually reclassified from low to high risk of fatal events as a result of prespecified cutoff points of inflammatory markers was not provided, and would help assess the clinical value of adding inflammatory biomarkers to conventional risk models. This is particularly important given the age and baseline risk of the population studied. The relevance of these results to younger, lower-risk populations will require further study.

Whiteley et al. investigated the potential of inflammatory markers to add to the prediction of functional outcomes in patients suffering an acute stroke [10]. Using a simple six-variable prognostic model as the reference, the researchers performed a detailed evaluation of the predictive value of inflammatory markers. This evaluation included assessing the global fit of the model, the ROC curve for discrimination between subjects with good or poor outcome, calibration to assess for the relationship between predicted and observed risk, and, most importantly, reclassification of patient risk. By the likelihood ratio statistic and by the ROC curve, addition of IL-6 (or a composite of IL-6, CRP, and white cell count) to the six-variable model significantly improved the prediction of outcomes. Similar quantitative relationships between IL-6 levels and outcome were evident in the studies included in their meta-analysis. However, because only 5% of patients were reclassified from an intermediate risk category to a category with>90% or<10% risk of poor outcome, the authors concluded that the value of adding inflammatory markers to prediction models was modest.

As the authors point out, only 60% of patients had inflammatory markers measured, and this population experienced milder strokes than the overall population, raising concerns about the applicability of the data to patients with more severe strokes. A significant number of patients had inflammatory markers drawn more than 1 week after presentation (median 13 days), confusing elevated markers at baseline with those emerging as a result of the complications of stroke (such as urinary tract infections). The number of patients reclassified from indeterminate to determinate risk categories is dependent upon the percent risk used to define low (<10%) or high (>90%) risk of poor outcome. Although IL-6 may have improved reclassification to a greater degree under less stringent criteria to define low and high risk (eg<20% and>80% respectively), the clinical utility of these categories of risk is uncertain.

## Two Apparently Different Conclusions

If the authors of the two studies have reached different conclusions on the utility of inflammatory markers, can, or should, the results of the two studies be reconciled? Sattar et al. used inflammatory markers to predict cardiovascular mortality in a large population without current infarctions, whereas Whitely et al. used inflammatory markers to predict functional outcomes in patients presenting with stroke. In the former, inflammatory markers provided insights into inflammation as a predictor or cause of life-threatening plaque rupture and new arterial occlusions, or the survival after such an event. In the latter, the relationship of inflammatory markers to the extent of neurological injury [11] and to comorbidity, rather than propensity to new arterial occlusions, was assessed. Thus the pathologies in the two studies are quite distinct. In addition, there are important differences in the populations being studied and the questions being posed.

In large populations with a relatively low incidence of disease, as in Sattar et al., a several-fold increase in risk can be associated with a substantial increase in the total number of events, and can therefore permit reclassification of patients from low to intermediate or high risk categories, yet potentially not alter the ROC curve C-statistics [6]. Where such reclassification of calculated risk (e.g., 5 year risk of fatal myocardial infarction) changes management by qualifying patients for primary prevention with medication such as aspirin or statins, a large number of fatal cardiovascular events may be prevented.

In the case of patients suffering from stroke, prognostic stratification could be used to determine optimal use of thrombolytic therapy or to decide on which patients require treatment with statins or other drugs. The denial of potentially life-saving treatment in these patients requires great confidence in the ability of the biomarker (in conjunction with preexisting risk predictors) to predict outcome. The expectations of predictive models in these circumstances are high in order to justify a denial of treatment. The expectations are harder still to meet when a relatively simple and robust predictive model using six clinical variables provides excellent discrimination of outcomes as demonstrated by Whiteley et al.

## Implications and Future Directions

The study by Sattar et al. will prompt reanalysis of the predictive value of IL-6, CRP, and other inflammatory markers in various patient populations. Clinicians may need to consider cardiovascular mortality, rather than total cardiovascular events, when combining inflammatory biomarkers with conventional Framingham criteria to calculate absolute risk. The study by Whiteley et al. will reassure clinicians that clinically meaningful prediction of outcomes after stroke is feasible using a simple clinical score. Further studies that include patients with more severe strokes will be needed to resolve the question of whether inflammatory markers have predictive value in this population.

## Abbreviations

CRP: C-reactive protein
IL-6: interleukin-6
ROC: receiver operating characteristic
